# Implementing Psychosocial Evidence-Based Practices in Mental Health: Are We Moving in the Right Direction?

**DOI:** 10.3389/fpsyt.2015.00051

**Published:** 2015-04-14

**Authors:** Alfonso González-Valderrama, Cristián Mena, Juan Undurraga, Carlos Gallardo, Pilar Mondaca

**Affiliations:** ^1^Early Intervention in Psychosis Program, Instituto Psiquiátrico “Dr. José Horwitz Barak”, Santiago, Chile; ^2^Facultad de Medicina, Universidad Finis Terrae, Santiago, Chile; ^3^Department of Psychiatry, Facultad de Medicina Clínica Alemana Universidad del Desarrollo, Santiago, Chile

**Keywords:** psychosocial interventions, evidence-based practice, guideline adherence, schizophrenia, mental health services

One of the main goals of research in health sciences is to provide clinically relevant information aimed at generating effective interventions in the patients’ care. Many ways to bring the new knowledge into daily practice have been implemented: clinical guidelines, reviews in journals, continuing medical education (CME), courses and seminars, easy access to online information, and others (1). However, there is a gap between evidence-based knowledge and clinical practice in different medical settings (2). A good example of these kind of difficulties is hand washing: It is a well-known evidence-based measure; however, it is difficult to implement: one-third of health-care workers do not incorporate this habit after learning a specific strategy (3).

Evidence-based interventions are even more difficult when we have to perform more complex actions like interventions in mental health especially in severe mental disorders. A recent review of the Cochrane Schizophrenia Group examined the efficacy of guideline implementation strategies in improving process (performance of health care providers) and patient outcomes in schizophrenia and related psychotic disorders (4). Authors compared guideline implementation strategies with usual care and, in addition, assessed the comparative efficacy of different guideline implementation strategies. The results were by far disappointing: only 5 of 867 studies met inclusion criteria and all evidence was assessed by the authors as “very low quality.” There was no effect in antipsychotic polypharmacy reduction and no impact in clinical symptoms, care satisfaction, drug attitude, or adherence rates. Only one study showed a significant effect in the proportion of patients screened for blood pressure and cholesterol, but not other cardiovascular risk factors (4). Besides, the studies did not include patient’s quality of life, which is an important outcome in the evaluation of interventions.

Furthermore, we can asseverate that mental health psychosocial interventions are one of the most complex issues when we put evidence into practice. Briand and Menear divided the evidence-based psychosocial interventions in three domains: 1) illness self-management, medication management, health promotion, and (or) psychological interventions; 2) social interventions such as social skills training or supported employment; and 3) service level interventions (5). These domains bring together several types of activities, with different objectives and methodologies. Therefore, the difficulties in the implementation of psychosocial evidence-based practices in mental health could be due to: technical characteristics of interventions (whether in relation to the strength of the evidence, complexity of the multidisciplinary treatments, differences in local contexts, and costs); characteristics of the health staff involved in its implementation (lack of access to knowledge, skills, personal attributes, and attitudes regarding changes); characteristics of the organizations and its openness to changes; leadership abilities by decision-makers; and, finally, ways of monitoring the implementation of evidence-based practices (6). These difficulties have been described in countries of high income, mainly North America (United States, Canada) and Europe. However, few reports have been made in countries of middle-high income.

Chile can be a concrete example of the described difficulties. Since 2000, The Ministry of Health has undertaken a mental health reform process with emphasis in a community model, focusing efforts in increasing the number of patients treated in mental health services. Among many advances, we can mention the creation of a National Plan of Mental Health and Psychiatry, an increase in human resources within mental health teams, an increase in mental services in primary care, and evidence-based treatment guidelines. Four guidelines aimed at treating first episode of schizophrenia, depression, bipolar disorder, and substance abuse in persons under age 20 have been developed. All of them suggest multiple psychosocial interventions. Unfortunately, at this moment, there is no systematic evaluation of their implementation and the quality or effectiveness of these interventions. This has been considered recently in the 2014 report of Chilean mental health system using the WHO–AIMS tool (7).

Lack of evaluation of guideline implementation is a common issue in mental health interventions as can be observed in the Chilean Schizophrenia Guideline (8). Treatment recommendations in this guideline include several psychosocial interventions: individual and family psychoeducation, cognitive behavior therapy (CBT), and/or other psychotherapies, social skills training, art therapy, supported employment, and recreational interventions. However, what it is written does not always work as expected. Pantoja et al. (9) assessed the quality of this clinical guideline through the AGREE instrument (10). The study showed that it has only an 11% of applicability; therefore, it is likely that it is not being properly applied in clinical practice, especially regarding psychosocial interventions. Once again, the phantom of the gap between the evidence and the practice remember us that it is essential to describe, to evaluate, and to analyze the real difficulties if we want to improve the performance of our daily clinical practice (11, 12).

Another difficulty found when implementing psychosocial interventions is cost. This is specially relevant in developing countries, where resources are scarce and should be allocated accordingly. Nonetheless, economic evidence is based mainly in cost-effectiveness studies regarding medications, with little attention given to psychosocial interventions (13, 14). Still, there is data supporting the use of combined treatment (i.e., pharmacologic and psychosocial interventions) as a cost-effective intervention in countries from the developing world such as Chile, Nigeria, Sri Lanka, and Thailand (15, 16). A recently published randomized controlled trial from China compared antipsychotics combined with psychosocial treatment and treatment as usual for patients with early stage schizophrenia (17). They found no significant differences in direct costs, indirect costs, and total costs between two groups (all *p*-values ≥0.556). In addition, they found that combined treatment was associated with significant higher quality-adjusted life year ratings than treatment as usual (*p* = 0.039). Moreover, there is evidence that linking healthcare providers to community resources is a cost-effective intervention that could improve health and well-being of people with long-term conditions, especially the mentally ill (18). This is relevant, as there are community resources not being considered when planning interventions in mental health.

Other developing countries probably share some of the difficulties described. We believe that it is essential to make improvements at different levels in order to advance in the applicability of the available evidence. Mental health teams must evaluate the local experiences and difficulties in the implementation of these guidelines and they must receive continuing education and training in their specific psychosocial interventions. Therefore, there should be free access manuals and assessment tools to mental health teams, especially in countries where mental health services are still in developing stage. Assessment of implementation guidelines and effectiveness measures must be included by policy makers. We have to include a user satisfaction assessment, if we want to improve our services in mental health. Finally, it is important to promote researches related to this topic looking for all kinds of innovations in the implementation of emerging evidence in many different psychosocial interventions.

It is a necessity to improve our daily practice with the best available evidence, specifically in the psychosocial interventions in mental health services. This issue is very important for mental health professionals, as it will be considered this year in the 5th European Conference on Schizophrenia Research where motto “Bridging Gaps – Improving Outcomes” will reinforce the importance of recognizing this gap that persists in the treatment of mental health disorders.

We would like to suggest to read again the invitation by Grol and Grimshaw: “If you would like to start tomorrow to change practice and implement evidence, prepare well: involve the relevant people; develop a proposal for change that is evidence based, feasible, and attractive; study the main difficulties in achieving the change, and select a set of strategies and measures at different levels linked to that problem; of course, within your budget and possibilities. Define indicators for measurement of success and monitor progress continuously or at regular intervals. And, finally, enjoy working on making patients’ care more effective, efficient, safe, and friendly …” (19).

Are we moving in the right direction?

## Conflict of Interest Statement

The authors declare that the research was conducted in the absence of any commercial or financial relationships that could be construed as a potential conflict of interest.
